# Molecular and Serological Characteristics of Avian Pathogenic *Escherichia coli* Isolated from Various Clinical Cases of Poultry Colibacillosis in Poland

**DOI:** 10.3390/ani12091090

**Published:** 2022-04-22

**Authors:** Jarosław Wilczyński, Dagmara Stępień-Pyśniak, Danuta Wystalska, Andrzej Wernicki

**Affiliations:** 1 Veterinary Diagnostic Laboratory Lab—Vet, 62-080 Tarnowo Podgórne, Poland; wilczynski@lab-vet.com.pl (J.W.); wystalska@lab-vet.com.pl (D.W.); 2 Department of Veterinary Prevention and Avian Diseases, Faculty of Veterinary Medicine, University of Life Sciences in Lublin, 20-612 Lublin, Poland; andrzej.wernicki@up.lublin.pl

**Keywords:** colibacillosis, APEC, *E. coli* serotypes, virulence genes

## Abstract

**Simple Summary:**

Infections caused by avian pathogenic *Escherichia coli* (APEC) represent a serious threat for poultry production and economic losses. These bacteria have a number of mechanisms that enable them to colonize various ecosystems. We analyzed *E. coli* from chicken and turkey broilers, laying hens, and broiler parents. In this work, we characterized virulence factors in APEC isolated from poultry in Poland. We also attempted to show a correlation between lesion severity, the presence of virulence genes, and the *E. coli* serogroup. The most prevalent serotype among *E. coli* isolates from all types of chickens was serotype O78, in contrast to isolates from turkeys, in which the prevalence of four serotypes, O1, O2, O18, and O78, was similar. Once the serotype was distinguished, it was possible to assign certain virulence characteristics to the isolate and to associate it with the symptoms present, as well as to track the frequency of occurrence. All of the eight virulence genes analyzed were most common in cases with severe, generalized disease processes. The results also confirmed that there is a great variety of APEC strains in the poultry population.

**Abstract:**

*Escherichia coli* infections are a major problem in modern poultry production. Avian pathogenic *E. coli* (APEC) strains have several mechanisms that enable them to colonize various ecosystems. In this study, 290 *E. coli* isolates were recovered from clinical cases of colibacillosis in chicken and turkey broilers and from laying and breeding hens. The samples were taken from organs with pathological changes suggesting colibacillosis. The lesions were assigned to three groups depending on their advancement, of which the largest (60% of the isolates) was group 3, with the most extensive changes. The most common serotype was shown to be O78 (14%). The most frequently detected gene among those tested was *iss*, while *papC* was the least prevalent. An analysis of the number of genes present per isolate revealed that the presence of four genes was the most common (22%), while only 1% of the strains tested had all eight genes. The most frequently detected genes for each serotype were *iss* and *iucD* for O78; *irp2* and *cvi/cva* for O1; *irp2*, *iucD*, and *iss* for O2, and *iss* and *iucD* for O8, for which the least frequent was *papC*. All O18 serotype strains had the *iss* gene, while none had the *vat* gene.

## 1. Introduction

Avian colibacillosis caused by *Escherichia coli* APEC strains is one of the major causes of economic losses in poultry production [1]. Changes in the course of colibacillosis may affect various systems, e.g., the respiratory, digestive, reproductive, or locomotor systems, or may occur locally, as in the case of yolk sac infections, omphalitis, dermatitis, cellulitis, or panophthalmitis. These are often secondary infections; in such cases, primary diseases are complicated by *E. coli* [2]. Losses due to APEC infections are associated with high mortality, ineffective therapy, low weight gains in meat-type birds, decreased laying, fertilization, and hatchability, and an increase in the number of carcasses or their parts discarded due to pathological changes [3,4,5,6,7,8]. There is considerable phylogenetic similarity between APEC strains and strains from extraintestinal pathogenic *E. coli* (ExPEC) infections in humans. Such strains have also been shown to share the same virulence genes [9,10].

Currently, there are 180 envelope O antigens, 60 ciliary H antigens, and 80 surface K antigens. Once the serotype is distinguished, it is possible to assign certain virulence characteristics to the isolate and to associate it with the symptoms present, as well as to track the frequency of occurrence. The most common serotypes associated with infections involving APEC have been shown to be O1, O2, O35, O36, and O78 [11,12,13]. Other serotypes have been found less frequently, and their prevalence is varied depending on the location of the outbreak and on the species, age, and utility type of the birds. In addition, many analyzed APEC isolates have been found to be ‘not typable’ [14].

O-specific antigens are components of lipopolysaccharides (LPSs) and are among the most important markers used in the diagnosis of APEC strains. They are identified by means of serological reactions or biosynthesis gene analysis [15]. They are a component of LPSs exposed on the bacterial cell surface and are recognized by immune system factors and bacteriophages. The primary function of O antigens is to protect the bacterial cell from complement components and other defense proteins such as BPI (bactericidal permeability-increasing protein) or defense peptides. The specificity of the O antigen is linked to adaptation to the environment and its variability to immune evasion mechanisms. Serotyping is used to determine the source of an outbreak and the pathogenicity of a strain. However, serotyping alone does not provide detailed knowledge of the virulence characteristics of the bacteria.

According to genetic criteria, the classification of *E. coli* as an APEC strain is associated with the presence of at least five of eight virulence genes [16]. The pathogenicity of APEC strains is facilitated by virulence factors represented by adhesins (encoded by *tsh* (temperature-sensitive hemagglutinin) and *papA* (Pap fimbrial major pilin protein)), protectins (encoded by *iss* (increased serum survival)), toxins (encoded by *astA* (enteroaggregative toxin), *vat* (vacuolating autotransporter toxin), and *cvi/cva* (colicin V plasmid operon genes), e.g., *cvi* (ColV immunity) and *cva* (ColV export)), and iron acquisition systems (encoded by *irp*2 (iron-repressible protein) and *iuc*D (aerobactin biosynthesis protein)). These genes are located on mobile structures, including plasmids, transposons, pathogenicity islands, and bacteriophages, which may occur singly or in groups [17].

Poultry colibacillosis, due to the course and nature of the lesions, is one of the most frequently diagnosed diseases in poultry production. Laboratory diagnostics of *E. coli* infections are based mainly on the culture tests of samples taken from pathologically changed organs. Then, the bacterial colonies are subjected to phenotypic evaluation, i.e., the analysis of basic biochemical properties and the determination of drug resistance. Unfortunately, routine diagnostics are not sufficient to determine pathogenic potential or to compare virulence characteristics between isolates from the same stock. Distinguishing potentially pathogenic *E. coli* from nonpathogenic *E. coli* isolates seems to be of paramount importance, especially in the diagnosis of polyetiological diseases, in which *E. coli* often complicates the primary disease. The presence of *E. coli* in examined material is not sufficient to assess the course and prognosis of the disease or to determine treatment. The pathogenic potential of isolated bacteria should be assessed in order to properly diagnose the disease.

Therefore, the aim of this study was to analyze clinical cases of colibacillosis and then to characterize the prevalence of serogroups and virulence-associated genes in avian pathogenic *Escherichia coli* isolated from various species and utility types of poultry in Poland. In addition, an attempt was made to determine the correlation between lesion severity, the presence of virulence genes, and the *E. coli* serogroup.

## 2. Materials and Methods

### 2.1. Anatomopathological Examination

The birds used in post-mortem examinations were submitted to the Veterinary Laboratory Diagnostic Lab-Vet Sp. z o.o. in Tarnowo Podgórne by veterinarians or poultry farmers for laboratory testing. Lesions in internal organs indicating colibacillosis were assessed, including the type, size, and frequency of lesions.

Only cases in which macroscopic lesions suggestive of colibacillosis were present in a majority of the examined birds representing a given flock were analyzed. The cases were divided into three groups based on the severity of the lesions according to the criteria described by Van Eck and Goren [18].

Group 1 comprised two or more necrotic foci located in the internal organs (liver, spleen, kidney, or pericardial sac), cloudiness of the air sacs, hyperemia of the yolks (in laying hens), and hyperemia in the navel area and yolk sac (in the case of chicks). Group 2 included a thin layer of fibrous exudate located on the serosa and internal organs (liver, spleen, kidney, pericardial sac, or air sacs), a cheese-like coating on the yolks, cloudy fluid in the oviduct (in layers), and altered hyperemia of the yolk sac (in chicks). Group 3 described a thick layer of fibrous exudate on the surface of internal organs (liver, spleen, kidney, pericardial sac, or air sacs) and serous membranes, coagulated eggs in the body cavity (in layers), and inflammatory lesions in the umbilical region and clotted masses in the yolk sac (in chicks).

### 2.2. Bacterial Strains

A total of 290 *E. coli* isolates were obtained from pathologically changed internal organs (heart, liver, joint cavity, air sac, and spleen) of dead birds with lesions typical of colibacillosis. According to local law (The Act of 15 January 2015 on the protection of animals used for scientific or educational purposes), permission from the Ethics Commission was not required for this type of sample collection (the sampling of dead birds). The isolates were derived from the following species and utility types of poultry: broiler chickens (111), broiler turkeys (75), laying hens (45), and broiler breeding hens (59). The samples for bacteriological analysis were collected with sterile swabs (MWE medical wire, Corsham, Wiltshire, England) and inoculated onto MacConkey agar (OXOID, Basingstoke, Hampshire, UK) and Columbia Agar with 5% defibrinated sheep blood (OXOID, Basingstoke, Hampshire, UK). The inoculated media were incubated at 37 ± 1 °C for 24 h under aerobic conditions. *E. coli* isolates pre-identified based on their morphology were further seeded onto Nutrient Agar (OXOID, Basingstoke, Hampshire, UK) for further analysis.

### 2.3. Identification of Isolates by Real-Time PCR

First, genomic DNA was extracted using a commercial Kylt DNA Extraction-Mix II (Kylt, AniCon Labor GmbH Emstek, Germany) according to the manufacturer instructions. Then, identification of the 290 isolates initially identified as *E. coli* was performed using a commercial Real-Time PCR Kylt *E. coli* assay (Kylt, AniCon Labor GmbH, Emstek, Germany) and a Rotor Gene Q cycler (Qiagen, Hilden, Germany). The *Escherichia coli* ATCC 25,922 and *Enterococcus faecalis* ATCC 29,212 reference strains were used as positive and negative controls.

### 2.4. Serotyping

The determination of O (somatic) antigens was carried out using a slide agglutination test with *E. coli* antisera (Biovac, Beaucouzé, France; Sifin, Berlin, Germany; Statens, Copenhagen, Denmark) according to the manufacturer instructions. The tests were performed for O1, O2, O18, O78, and O8 somatic antigens, which are the most common in poultry [11,19].

### 2.5. Virulence Genotyping

All 290 isolates were screened for the presence of eight virulence genes (*iss*, *irp2*, *iucD*, *cvi/cva*, *astA*, *papC*, *tsh*, and *vat*) by PCR amplification with the primers and conditions described in Table 1. Uniplex PCR was carried out for the *iucD*, *cvi/cva*, *papC*, *tsh*, *vat*, and *astA* genes. PCR was performed in a total volume of 20 µL containing 1 µL of DNA as a template; 2 µL of reaction buffer (10x) with 25 mM MgCl_2_; 0.2 µL of Viva Taq DNA polymerase (5 U/µL) (Novazym, Poznan, Poland); 1 µL each of two primers (10 pmol/µL; Genomed, Warsaw, Poland); 0.8 µL of 5 mM dNTPs MIX; and 14 µL of MilliQ water. Duplex PCR was carried out for the *iss* and *irp2* genes. The composition of the reaction mixture was as for uniplex PCR, except that 0.5 μL of each primer was added, and the amount of water was reduced. The temperature and time conditions were as follows: 1 cycle at 94 °C for 2 min; 30 cycles, each at 94 °C for 30 s; annealing of the primers at the temperatures given in Table 1 for 30 s and at 72 °C for 45 s; and a final extension at 72 °C for 8 min [19]. The PCR products were analyzed by electrophoretic separation on 1.5% agarose gel for 40–60 min at a voltage of 110 V in a 0.5% TBE solution. The amplicons were stained with GelView dye (Novazym, Poznan, Poland). The PCR reaction products were interpreted by comparing them to size standards and positive controls. *E. coli* 17-2 (serotype O:3, H:2) for *astA* and *irp2*; *E. coli* IMT 2467 (serotype O1:K1) for *papC*; *E. coli* IMT 663 (serotype O:78, K:80) for *tsh* and *iucD*; and *E. coli* IMT 5155 (serotype O:2) for *vat*, *cvi/cvaC*, and *iss* were used as positive controls. All positive controls for the virulence factors were obtained courtesy of AniCon Labor GmbH (AniCon Labor GmbH, Emstek, Germany). Nuclease-free water was used as a negative control.

### 2.6. Statistical Analysis

For statistical analysis, the significance of differences in the number of detected *E. coli* strains in individual poultry species and types was examined using the chi-square test. Statistical analyses of the remaining results were performed using quasi-Poisson regression. All of the statistical analyses were performed with the use of the R environment (R Core Team; A language and environment for statistical computing; Foundation for Statistical Computing, Vienna, Austria, 2021).

## 3. Results

### 3.1. Anatomopathological Examination

According to the adopted criteria, 60% of all the analyzed cases were assigned to group 3, in which the post-mortem lesions were determined to be the most severe, indicating an advanced course of the disease with generalized infection. This group included as many as 71% of all the cases from broiler breeding hens, while the lowest percentage, 48%, was obtained from cases in broiler chickens. Group 2 accounted for 26% of all the cases supplied for the study, among which cases from broiler chickens predominated (38%). Group 1, the least numerous, comprised 14% of all the cases studied. The percentage of isolates with anatomopathological lesions characteristic of group 1 was similar for all bird species and types, ranging from 12% to 16%. Detailed results of anatomopathological lesions of varying degrees of severity in the course of colibacillosis in each of the poultry species and types are presented in Table 2. The severity of lesions in internal organs indicating colibacillosis in different species and utility types of poultry are shown in Table S1. Examples of anatomopathological lesions representing different degrees of severity can be found in Figures S1–S6.

### 3.2. Identification of Isolates by Real-Time PCR

All 290 isolates were correctly identified as *E. coli* using a commercial Kylt *E. coli* Real-Time PCR assay (Kylt, AniCon Labor GmbH, Emstek, Germany). The analysis of the reaction was preceded by the analysis of an internal control, whose curve was read in the HEX channel, while the result for the specificity of *E. coli* identification was read in the FAM channel. The sample was considered positive if a characteristic amplification curve was visible in the FAM and HEX channels.

### 3.3. Serotyping

Among all *E. coli* isolates tested, 93 (32%) reacted positively with the sera used. The remaining 68% of the isolates did not show a positive reaction with the sera. The highest percentage of positive results (38%) was obtained for *E. coli* isolated from commercial laying hens, while the fewest seropositive isolates (23%) were found in broiler chickens. O78 was the dominant serotype, accounting for 14%, while serotype O8 was the least frequently identified (2%). The other serotypes (O1, O2, and O18) occurred with similar frequency, ranging from 4% to 6%. In broiler chickens, serotypes O78 (8%) and O18 (8%) were the most prevalent. Among all *E. coli* isolates from broiler chickens, only 1% were *E. coli* isolates belonging to serotype O2. In the case of broiler turkeys, serotypes O1, O2, O18, and O78 occurred with a similar frequency of 7–9%. Only serotype O8 was representative of just 1% of all the strains isolated from broiler turkeys. Only three serotypes were found in broiler breeding hens (O78, O1, and O2), of which O78 was dominant (34%), while serotypes O8 and O18 were absent. The predominant serotype in commercial laying hens was serotype O78 with 29% frequency. No O1 serotype was found among the isolates obtained from commercial laying hens. Detailed results of the serological tests are presented in Table 3.

### 3.4. Prevalence of Virulence-Associated Genes

Among virulence factors, the phagocytosis protection factor, encoded by the *iss* gene, was present in as many as 89% of the strains isolated from all species and utility types of poultry. A significant percentage of positive results was also obtained for the *iucD* (75%) and *irp2* (60%) genes encoding siderophores, which are responsible for the acquisition of iron ions in the host system. The gene detected least frequently in the tested strains was *papC* (13%), which is responsible for coding fimbria P adhesins, a factor of the colonization in extraintestinal infections and the stimulation of cytokine production by T lymphocytes. Moreover, among *E. coli* isolated from broiler turkeys and commercial laying hens, a significant number of positive results (51–68%) was observed for the *cvi/cva* (colicin V) gene, a factor facilitating colonization, and the *tsh* gene encoding hemagglutinin, which is responsible for erythrocyte agglutination. Compared to other poultry types, among isolates from breeding hens there was a relatively high number of positives for the *vat* gene (56%) encoding Vat toxin, which causes the intracellular vacuolization of host cells. Detailed data on the results pertaining to genetic determinants of virulence in different poultry species and types are presented in Table 4.

### 3.5. Gene Profile Results

The results concerning the occurrence of pathogenicity genes were used to analyze the potential virulence of the 290 *E. coli* isolates. The isolates were classified into nine virulence groups according to the number of genes they possessed: Group 1—no genes found; Group 2—one gene present; Group 3—two genes present; Group 4—three genes present; Group 5—four genes present; Group 6—five genes present; Group 7—six genes present; Group 8—seven genes present; and Group 9—eight genes present.

Most strains had a set of four, five, or six virulence genes in their genotype, accounting for 22%, 19%, and 18% of the isolates, respectively. In Groups 5 (4/8 genes) and 6 (5/8 genes), the most isolates were from commercial laying hens and broiler chickens, while in Group 7 (6/8 genes) the most isolates were obtained from commercial laying hens and broiler turkeys. Fewer isolates were assigned to Groups 3 (2/8 genes) and 8 (7/8 genes)—only 6% each. Group 1, which comprised strains that had no virulence gene in their genotypes, contained 6% of all the isolates tested. Isolates whose genotype had a full set of virulence genes were found only in broiler turkeys (5%) and accounted for 1% of all *E. coli* tested. Details of the relationship between the number of virulence genes found per isolate and the species and utility type of the poultry are presented in Table 5.

According to the definitions that have been applied by some authors [20,21] APEC strains are *E. coli* isolated from clinical cases of avian colibacillosis with five or more virulence genes. Assuming these criteria, 43% of all the strains tested were APEC, with comparable results in different groups, e.g., 42% in broiler chickens, 48% in broiler turkeys, 47% in commercial laying hens, and 37% in broiler breeding hens. The *iucD* gene was found most frequently and the *papC* gene least frequently. Details of the prevalence of APEC strains according to the above definition are shown in Table 6.

### 3.6. The Prevalence of Virulence Genes in Individual Types of Poultry

**Figure 1:**
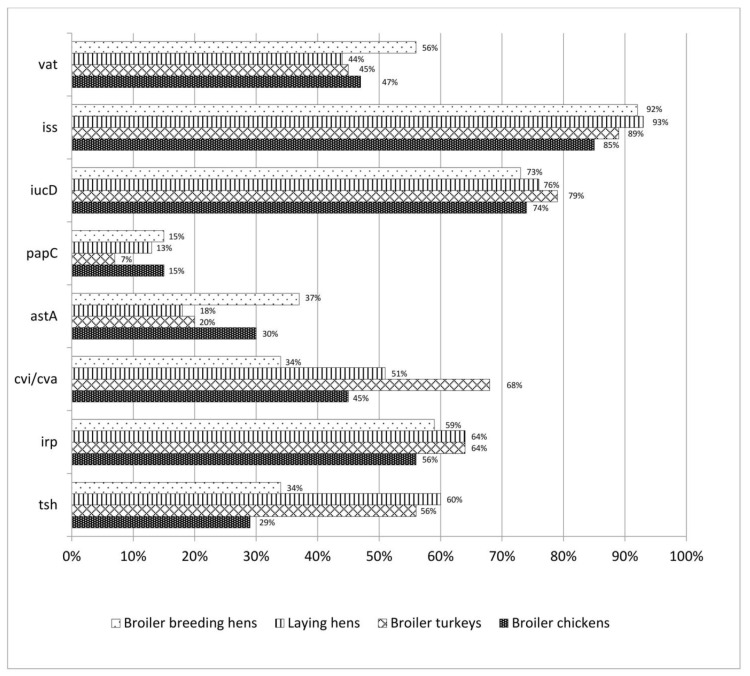
Prevalence of individual virulence genes among *E. coli* strains depending on the species and utility type of poultry.

The *papC* gene was most frequently found in broiler chickens and broiler breeding hens (in 15% each) and least frequently in broiler turkeys (7%). The *tsh* gene was most frequently found in commercial laying hens (60%) and least frequently in broiler chickens (29%). The *cvi/cva* gene was confirmed most frequently in strains from broiler turkeys (68%). Half as many strains carrying the *cvi/cva* gene originated in broiler breeding hens (34%). The number of isolates carrying the *irp2* gene was comparable in all poultry types. The highest percentage of this gene was observed in *E. coli* isolated from commercial laying hens and broiler turkeys, with 64% each. The results obtained for the *iucD* gene were comparable and above 70%. However, the most isolates possessing this gene were isolated from broiler turkeys (79%). For the *iss* gene, the results in all groups were about 90%. The highest percentage of *E. coli* isolates (93%) possessing this gene was obtained from commercial laying hens and the lowest from broiler chickens (85%). For the *astA* gene, significant differences were observed depending on the poultry type. Among strains derived from broiler breeding hens, the *astA* gene was found in as many as 37%, while it was present in only 18% of the strains isolated from commercial laying hens (the lowest frequency). The results obtained for the *vat* gene were comparable at about 50%. The highest number of strains possessing this gene came from broiler breeding hens (56%) and the lowest from commercial laying hens (44%). Detailed data on the frequency of gene occurrence in individual poultry species and utility types are presented in Figure 1.

### 3.7. Serotyping in Relation to the Presence of Virulence Genes

An analysis of the results obtained for virulence genes in relation to *E. coli* serotypes revealed that, for serotype O78, the most frequently detected genes were *iss* (98%) and *iucD* (93%). In the case of serotype O1, the results were similar to those for serotype O78, but additionally a relatively high percentage of strains carried the *irp2* (79%) and *cvi/cva* (71%) genes. Furthermore, the *irp2*, *iucD*, and *iss* genes were found in 100% of the O2 serotype strains. Additionally, *tsh*, *cvi/cva*, and *vat* genes were detected in 92% of the strains. Interestingly, no *papC* gene was found for this serotype. Serotype O8 was associated with a relatively low number of detected genes. The most frequently found genes were *iss* (69%) and *iucD* (56%), while the least commonly detected was the *papC* gene (19%). All strains belonging to serotype O18 had the *iss* gene, while none of them had the *vat* gene. Details of the relationship between the genes found and the *E. coli* serotypes are shown in Table 7.

### 3.8. Pathological Changes and the Presence of Virulence Genes

**Figure 2:**
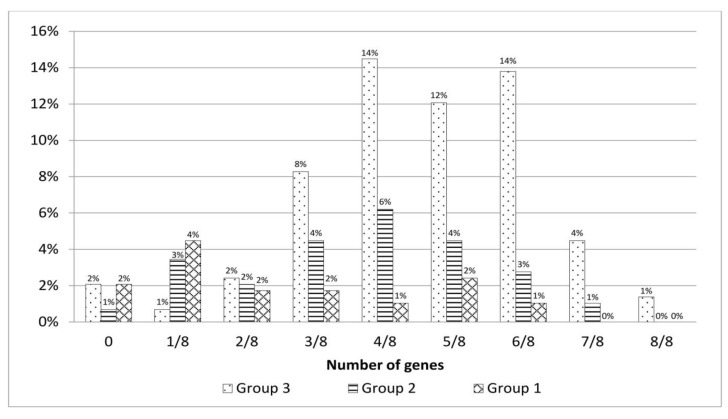
The presence of virulence genes in *E. coli* depending on the advancement of pathological changes.

The percentages of *E. coli* isolates in which no virulence genes, or only one or two, were found were comparable for all three groups categorizing the severity of anatomopathological lesions. However, for clinical cases from which isolates with three or more virulence genes were derived, a significantly higher number of cases was assigned to the group with the most severe lesions during post-mortem examination (group 3). There was also a low percentage of virulence gene positivity in group 1 of the post-mortem lesions of less than 2% (Figure 2).

## 4. Discussion

The main problem in the routine diagnosis of colibacillosis is to distinguish between pathogenic and nonpathogenic *E. coli* isolates. It is difficult to state unequivocally which *E. coli* strain one is dealing with on the basis of the basic phenotypic characteristics observed during routine diagnostic tests. Therefore, it is important to comprehensively evaluate the characteristics of APEC strains isolated from cases of colibacillosis. Among the many serotypes identified in clinical *E. coli* isolates [22], serotypes O78, O1, and O2 are the most frequently detected among putative APEC isolates recovered from cases of colibacillosis [11,12,13].

In our study, we found that the dominant serotype was O78, which was also predominant in the group of strains obtained from commercial laying hens and reproductive hens. Similar results have been obtained for strains isolated from broiler chickens in Korea [23] and Egypt [24], where the percentage of *E. coli* strains with serotype O78 was the highest in both countries at 20%. It is important to note that serotypes O78 and O18 were the most common serotypes found in broiler chickens in our study.

In contrast to these data, research conducted in Albania [25] showed that serotypes O8 and O86 were the predominant serotypes isolated from broiler chickens. In our study, serotype O8 accounted for only 2% of all *E. coli* isolates tested and only 3% of isolates from broiler chickens. Barbieri et al. [26], who studied *E. coli* isolated from clinical cases of colibacillosis in broiler chickens in Brazil, noted that serotype O78 was the predominant serotype, accounting for 23% of the strains tested, while strains from broiler chickens and commercial laying hens in Germany belonged mainly to serotype O2 (28.7%) [19]. Giovanardi et al. [27] published a study comparing the prevalence of specific *E. coli* serotypes isolated from colibacillosis cases in different broiler turkey flocks. They observed that serotype O78 was the predominant serotype in two of the three flocks, while serotype O2 was predominant in the third. These data are partially consistent with the results obtained in our study, where the predominant serotypes in turkeys were O2, O1, O18, and O78. In cases of APEC isolates from commercial laying hens in Belgium [28] and Poland (our study), serotype O78 proved to be the dominant serotype. It is also worth noting the presence of a significant percentage of APEC strains that did not show positive reactions in the slide agglutination test. Similar data were obtained in Germany [19], Albania [25], and Egypt [24], with 50.4%, 79%, and 33.3% nonserotyped strains, respectively.

In the last few decades, many authors have addressed issues related to the mechanisms of pathogenesis and epidemiology of APEC strains [13,19,29,30]. Numerous studies have aimed to find markers making it possible to differentiate strains that are part of the microbiota from pathogenic ones. Many authors emphasize the existence of APEC strains as a potential source of human infections with extraintestinal *E. coli* strains [31,32]. The present study focused on eight genes determining virulence in APEC strains: *papC*, *tsh*, *iucD*, *cvi/cva*, *iss*, *irp2*, *astA*, and *vat*. The data showed that *iss* was the most frequently found gene, followed by *iucD* and *irp2*. Similar results were obtained in Germany by Ewers et al. [19], who found a relatively high percentage of APEC strains possessing the *iss* (82.7%) and *iucD* (78%) genes. Comparable results were obtained for less frequently detected genes, such as *astA* (20%) and *papC* (22.7%). A slightly lower percentage of these genes (*iss*, 53.09%; *iucD*, 40.74%) among 129 APEC strains from breeding hens and broiler chickens was observed in Albania [25]. Nevertheless, these were the genes most frequently identified by these authors, in contrast to the *vat* (8.64%) and *cvi/cva* (8.65%) genes, which were the least prevalent. Similar results were obtained in Brazil in a study of 225 *E. coli* isolates from cases of air sac inflammation in broiler turkeys. They showed the presence of the *iss* gene in 93% of the isolates and *iucD* in 67%, while the *papC* gene was found in 15% and the *astA* gene in 17% of strains [33]. Mohamed et al. [34] also found that the most frequent gene was *iss* (72.2%), whereas the *papC* gene had a higher frequency (33.3%) than in our study and the one cited above.

A study conducted in Japan [35] comparing the prevalence of virulence genes in *E. coli* isolates from clinical cases in hens and from hens not showing signs of disease showed that the *iss* gene was more prevalent among isolates from birds showing clinical signs of colibacillosis. The *papC* gene had the lowest prevalence in isolates from hens with colibacillosis (24%), and it was not found in isolates from healthy hens.

Arabi et al. [20], after studying *E. coli* from clinical cases of colibacillosis in broiler chickens, proposed a definition of APEC strains as those possessing more than five virulence genes. Based on this definition, the authors classified only 26.7% of the isolates as APEC strains. Among these strains, the most prevalent was *iss* (96.4%), while the least frequent was *cva/cvi* (14.2%). Adopting the above criteria, our study showed that as many as 43% of the examined isolates could be classified as APEC strains. They most often possessed the *iucD* (100%), *iss* (97%), and *irp2* (92%) genes, while the least prevalent in these isolates were *papC* (25%) and *astA* (39%).

Similar criteria were adopted by authors from Korea [21], who compared the occurrence of virulence genes in APEC strains isolated from clinical cases of colibacillosis and from a hatchery environment. The authors showed that isolates from the hatchery environment most frequently carried the *iss* gene but at a relatively low level of 15%. The *iucD*, *tsh*, *vat*, and *cvi/cva* genes were not found in this group of strains. Among *E. coli* strains fulfilling the APEC criteria, the highest percentage was obtained from laying hens (31.3%), followed by broiler chickens (14%), and in the case of broiler breeding hens only 2.7%. In our study, the highest number of APEC strains was noted in broiler turkeys (48%), followed by commercial laying hens (46%), broiler chickens (42%), and broiler breeding hens (37%).

A study in the United States [36] comparing *E. coli* isolated from clinical cases of colibacillosis with isolates from fecal samples of healthy birds showed a higher prevalence of virulence genes among strains from the clinical cases compared to nonclinical strains (from feces). For example, the *iss* gene was found in 82.7% of the strains from diseased birds compared to just 18.3% of the strains isolated from healthy birds, and the *cvaC* gene was present in 67.4% of the strains from diseased birds and only 9.6% from healthy birds. Similarly, the *papC* gene was present in 40.4% of the isolates from diseased birds compared with 9.6% of healthy ones.

An analysis of the correlation of virulence genes and individual serotypes showed that all the *E. coli* isolates of serotypes O18 and O2 possessed the *iss* gene. This gene was also predominant in *E. coli* isolates belonging to serotype O78. Characteristically, the *papC* gene, whose frequency was the lowest, was present in less than half of the O18 serotype strains, while none of the O2 serotype strains possessed this gene.

Comparable results were obtained in research by Yaguchi et al. [35], in which 100% of the strains belonging to serotypes O2 and O78 possessed the *iss* gene. Similarly, in a study in Germany [19], the *iss* gene was present in 95.5% of the strains belonging to serotype O78 and 86% of the strains of serotype O2. In contrast, 100% of the O1 serotype strains possessed the *irp2* gene. In addition, in the study cited above, the *papC* gene had a relatively high prevalence in serotype O1 (55.6%), while in our study only a small percentage of the O1 serotype strains possessed the *papC* gene. Similar results were obtained by Shtylla et al. [25], who showed that 100% of the APEC strains belonging to serotype O78 possessed the *iss*, *irp2*, and *iucD* genes. Additionally, 50% of strains of this serotype possessed the *papC* gene. On the other hand, serotype O2 showed the highest prevalence of the *tsh*, *iucD*, and *iss* genes (100% for each).

Given that the strains we studied were isolated from pathologically altered organs and possessed specific virulence characteristics, it seems likely that they are associated with the pathological changes observed in the course of the various forms of colibacillosis from which the materials for analysis were obtained. This was also indicated by the number of genes per strain isolated from the most advanced cases of colibacillosis.

An analysis of the relationship between the severity of anatomopathological changes and the number of virulence genes among the APEC strains revealed that the strains isolated from cases assigned to the third group of anatomopathological changes (the most severe) had the highest number of these genes, most often 4–6 genes per APEC strain. In contrast, strains isolated from the cases belonging to group 1, with the least severe changes, had the lowest percentage of these genes. A single gene was usually found in such isolates.

Although the results indicated that *E. coli* isolates from advanced cases of colibacillosis possess several virulence genes simultaneously and represent specific serotypes characteristic of APEC strains, interpretation may nevertheless be difficult. First, there is no single virulence gene or set of such genes definitively associated with APEC. Secondly, *E. coli* isolates from nonclinically symptomatic birds may carry virulence genes as well. Thirdly, even if a strain possesses several virulence genes, they may not all be ‘active’. Nevertheless, the presence of ‘silent genes’ increases the risk that they will be activated by factors conducive to the occurrence of the disease, such as environmental or infectious factors acting on the avian immune system or host–pathogen interactions [37,38]. In addition, the virulence genes analyzed were located on genetic mobile elements, which may allow avirulent strains or those causing less severe lesions (strains with a lower virulence gene load) to become virulent, e.g., in subsequent generations of birds in a given poultry house.

In routine diagnostics, drug susceptibility testing should be considered in addition to characterization of the virulence of isolates, as APEC isolates are often multi-resistant [38].

In addition to serotyping, detection of the presence of virulence factors, and antimicrobial susceptibility analysis, *E. coli* isolates can be further characterized in terms of toxigenicity, cell attachment, invasiveness, hemagglutination, phage typing, virulence typing, plasmid profiling, and phylogenetic typing [15] to confirm the role of APEC strains (possessing specific virulence genes) in pathogenesis. Unfortunately, such studies require more financial resources and are often beyond the reach of routine diagnostics.

## 5. Conclusions

The most prevalent serotype among *E. coli* isolates from all types of chickens was serotype O78, in contrast to isolates from turkeys, in which the prevalence of four serotypes (O1, O2, O18, and O78) was similar. Among the analyzed *E. coli* isolates, the *iss* and *iucD* genes were most frequent, and the *papC* gene was the least frequent. Among all the serotyped strains, the *iss* and *iucD* genes were the most frequent. All of the eight virulence genes analyzed were most prevalent among the cases assigned to group 3 of the anatomopathological changes, indicative of a generalized disease process, in the post-mortem examination. The results also confirmed that there is a great variety of APEC strains in the poultry population. This raises the question of whether there is a viable method to reduce this pathogen in poultry, e.g., immunoprophylaxis with commercial vaccines. Studies carried out on virulence characteristics are the basis for the appropriate selection of strains for autogenic vaccines or bacterins.

## Figures and Tables

**Table 1:** Oligonucleotide sequences used in PCR reactions.

Gene	Primer	Primer Sequence	Annealing Temperature	Product Size (bp)	Locus
*astA*	EAST-1s	TGCCATCAACACAGTATATCC	54 °C	111	135–155
	EAST-1as	TAGGATCCTCAGGTCGCGAGTGACGGC			219–245
*papC*	PAPCs	TGATATCACGCAGTCAGTAGC	59 °C	501	1284–1304
	PAPCas	CCGGCCATATTCACATAA			1784–1767
*tsh*	TSHs	ACTATTCTCTGCAGGAAGTC	54 °C	824	132–151
	TSHas	CTTCCGATGTTCTGAACGT			955–937
*v* *at*	VATs	TCCTGGGACATAATGGTCAG	59 °C	978	1076–1095
	VATas	GTGTCAGAACGGAATTGT			2056–2038
*cva AB* *cvi/cvaC*	CVA1	TGGTAGAATGTGCCAGAGCAAG	65 °C	1181	10,745–10,764
	CVA2	GAGCTGTTTGTAGCGAAGCC			11,925–11,904
*irp2*	IRP-1	AAGGATTCGCTGTTACCGGAC	58 °C	413	22–42
	IRP-2	TCGTCGGGCAGCGTTTCTTCT			434–416
*iss*	ISSa	ATGCAGGATAATAAGATGAAA	58 °C	309	1–21
	ISSas	CTATTGTGAGCAATATACA			309–291
*iucD*	IucD-a	ACAAAAAGTTCTATCGCTTCC	58 °C	693	239–259
	IucD-as	CCTGATCCAGATGATGCTC			913–931

**Table 2:** Severity of anatomopathological lesions (divided into groups) in the course of colibacillosis in species and utility types of poultry—*n* (%).

Species and Utility Type of Poultry	Group 1	Group 2	Group 3	Total
Broiler chicken	16 (14)	42 (38)	53 (48)	111 (100)
Broiler turkey	12 (16)	13 (17)	50 (67)	75 (100)
Layer hen	7 (16)	10 (22)	28 (62)	45 (100)
Broiler breeding hen	7 (12)	10 (17)	42 (71)	59 (100)
Total	42 (14)	75 (26)	173 (60)	290 (100)

**Table 3:** Results of serological tests of *E. coli* strains showing positive reactions with diagnostic sera.

Species and Utility Type of Poultry	O1 *n* (%)	O2 *n* (%)	O8 *n* (%)	O18 *n* (%)	O78 *n* (%)	Total *n* (%)
Broiler chicken *n* = 111	4 (4)	1 (1)	3 (3)	9 (8)	9 (8)	26 (23)
Broiler turkey *n* = 75	7 (9)	7 (9)	1 (1)	6 (8)	5 (7)	26 (35)
Layer hen *n* = 45	0	2 (5)	1 (2)	1 (2)	13 (29)	17 (38)
Broiler breeding hen *n* = 59	2 (4)	2 (4)	0	0	14 (34)	18 (31)
Total *n* = 290	13 (4)	12 (4)	5 (2)	16 (6)	41 (14)	93 (32)

**Table 4:** Results of analysis of the presence of virulence genes in all tested *E. coli* isolates.

Species and Utility Type of Poultry	*papC* *n* (%)	*tsh* *n* (%)	*irp2* *n* (%)	*iucD* *n* (%)	*cvi/cva* *n* (%)	*iss* *n* (%)	*astA* *n* (%)	*vat* *n* (%)
Broiler chicken *n* = 111	17 (15)	32 (29)	62 (56)	82 (74)	50 (45)	94 (85)	33 (30)	52 (47)
Broiler turkey *n* = 75	5 (7)	42 (56)	48 (64)	59 (79)	51 (68)	67 (89)	15 (20)	34 (45)
Layer hen *n* = 45	6 (13)	27 (60)	29 (64)	34 (76)	23 (51)	42 (93)	8 (18)	20 (44)
Broiler breeding hen *n* = 59	9 (15)	20 (34)	35 (59)	43 (73)	20 (34)	54 (92)	22 (37)	33 (56)
Total *n* = 290	37 (13)	121 (42)	174 (60)	218 (75)	144 (50)	257 (89)	78 (27)	139 (48)

**Table 5:** Distribution of numbers of virulence genes in *E. coli* isolates obtained from each species and utility type of poultry.

Species and Utility Type of Poultry	Division into Groups According to Number of Genes								
	Group 1 (0/8)	Group 2 (1/8)	Group 3 (2/8)	Group 4 (3/8)	Group 5 (4/8)	Group 6 (5/8)	Group 7 (6/8)	Group 8 (7/8)	Group 9 (8/8)
Broiler chicken	9 (8)	9 (8)	9 (8)	11 (10)	26 (23)	27 (24)	18 (16)	2 (2)	0
Broiler turkey	5 (7)	9 (12)	2 (3)	8 (11)	15 (20)	10 (13)	15 (20)	7 (9)	4 (5)
Layer hen	2 (4)	2 (4)	4 (9)	5 (11)	11 (24)	9 (20)	9 (20)	3 (7)	0
Broiler breeding hen	0	5 (8)	3 (5)	18 (31)	11 (19)	9 (15)	9 (15)	4 (7)	0
Total	16 (6)	25 (9)	18 (6)	42 (14)	63 (22)	55 (19)	51 (18)	16 (6)	4 (1)

**Table 6:** Distribution of the presence of virulence genes among APEC strains (with at least 5 genes) in different species and utility types of poultry.

Species and Utility Type of Poultry	*papc* *n (%)*	*tsh* *n (%)*	*irp2* *n (%)*	*iucd* *n (%)*	*cvi/cva* *n (%)*	*iss* *n (%)*	*astA* *n (%)*	*vat* *n (%)*
Broiler chicken *n* = 47	15 (32)	20 (43)	42 (89)	47 (100)	33 (70)	44 (94)	21 (45)	36 (77)
Broiler turkey *n* = 36	5 (14)	32 (89)	36 (100)	36 (100)	35 (97)	36 (100)	11 (31)	30 (83)
Layer hen *n* = 21	3 (14)	20 (95)	21 (100)	21 (100)	19 (90)	20 (95)	4 (19)	12 (57)
Broiler breeding hen *n* = 22	9 (41)	15 (68)	17 (77)	22 (100)	15 (68)	22 (100)	13 (59)	14 (64)
Total *n* = 126 (43)	32 (25)	87 (69)	116 (92)	126 (100)	102 (81)	122 (97)	49 (39)	92 (73)

**Table 7:** The presence of virulence genes in *E. coli* serotypes.

Serotype	*papc* *n (%)*	*tsh* *n (%)*	*irp2* *n (%)*	*iucd* *n (%)*	*cvi/cva* *n (%)*	*iss* *n (%)*	*astA* *n (%)*	*vat* *n (%)*
**O1**	1 (7)	8 (57)	11 (79)	11 (79)	10 (71)	13 (93)	6 (43)	9 (64)
**O2**	0	11 (92)	12 (100)	12 (100)	11 (92)	12 (100)	2 (17)	11 (92)
**O8**	3 (19)	6 (38)	8 (50)	9 (56)	7 (44)	11 (69)	4 (25)	6 (38)
**O18**	2 (40)	1 (20)	3 (60)	4 (80)	1 (20)	5 (100)	1 (20)	0
**O78**	3 (7)	19 (46)	26 (63)	38 (93)	17 (41)	40 (98)	13 (32)	17 (41)

## Data Availability

The data presented in this study are contained within the article or Supplementary Material.

## Supplementary Materials

The following supporting information can be downloaded at: https://www.mdpi.com/article/10.3390/ani12091090/s1. Table S1: Severity of lesions in internal organs indicating colibacillosis in different species and utility types of poultry; Figure S1: Congestion of the umbilical region in a 2-day-old broiler chicken (Group 1 anatomopathological lesions); Figure S2: Cloudiness of air sacs in a 4-week-old broiler turkey (Group 1 anatomopathological lesions); Figure S3: Thin layer of fibrous exudate on the surface of the liver and pericardial sac in a 3-week-old broiler chicken (Group 2 anatomopathological lesions); Figure S4: Thin layer of fibrous exudate on the surface of the liver and pericardial sac in a 12-week-old laying hen (Group 2 anatomopathological lesions); Figure S5: Coagulated egg masses in the body cavity of a 30-week-old laying hen (Group 3 anatomopathological lesion); Figure S6: Thick layer of fibrous exudate on the pericardial sac in a 4-week-old broiler turkey (Group 3 anatomopathological lesions).
