# Supplementary material for: Molecular and Serological Characteristics of Avian Pathogenic Escherichia coli Isolated from Various Clinical Cases of Poultry Colibacillosis in Poland
Source: Animals (Basel). 2022 Apr 22;12(9):1090. doi: 10.3390/ani12091090 (PMC9106014; doi:10.3390/ani12091090)
Supplement: Supplementary file 1 [file animals-12-01090-s001.zip › Table S1.pdf]

Table S1. Severity of lesions in internal organs indicating colibacillosis in different species and utility types of poultry. These data correspond to Table 2 in the main text**.**

Case number	Species and utility type of poultry	Pericardium	Air sacks	Peritoneum	Liver	Joints	Spleen	Ovary	Yolk sac	Cellulitis
1	Laying hens	2	2	2	2	-	2	-	-	-
2	Laying hens	2	2	2	2	-	2	-	-	-
3	Broiler chickens	-	-	-	-	-	-	-	3	-
4	Broiler chickens	-	-	-	-	-	-	-	3	-
5	Laying hens	3	3	3	3	-	3	-	-	-
6	Laying hens	3	3	3	3	-	3	-	-	-
7	Laying hens	3	3	3	3	-	3	-	-	-
8	Laying hens	3	3	3	3	-	3	-	-	-
9	Laying hens	3	3	3	3	-	3	3	-	-
10	Laying hens	3	3	3	3	-	3	3	-	-
11	Laying hens	3	3	3	3	-	3	3	-	-
12	Laying hens	3	3	3	3	-	3	3	-	-
13	Laying hens	3	3	3	3	-	3	3	-	-
14	Broiler chickens	-	-	-	-	-	-	-	2	-
15	Broiler chickens	-	-	-	-	-	-	-	2	-
16	Broiler chickens	-	-	-	-	-	-	-	3	-

17	Broiler chickens	-	-	-	-	-	-	-	1	-
18	Broiler turkeys	1	1	-	-	-	-	-	-	-
19	Broiler turkeys	1	1	-	-	-	-	-	-	-
20	Broiler turkeys	3	3	3	3	-	3	-	-	-
21	Broiler breeding hens	3	3	3	-	-	-	-	-	-
22	Broiler breeding hens	2	2	2	-	-	-	-	-	-
23	Broiler breeding hens	3	3	3	3	-	3	-	-	-
24	Broiler chickens	2	2	2	2	2	2	-	-	2
25	Broiler chickens	3	3	3	3	-	3	-	-	-
26	Broiler breeding hens	1	1	1	1	-	1	1	-	-
27	Laying hens	-	-	1	1	-	1	-	-	-
28	Broiler chickens	-	1	1	-	-	-	-	-	-
29	Broiler chickens	3	3	3	3	-	-	-	-	-
30	Broiler turkeys	3	3	3	-	-	-	-	-	-
31	Broiler turkeys	2	2	2	-	-	2	-	-	-
32	Laying hens	3	3	3	3	-	3	3	-	-
33	Broiler turkeys	3	3	3	3	-	3	-	-	-

34	Broiler turkeys	3	3	3	3	-	3	-	-	-
35	Broiler chickens	3	3	3	3	-	-	-	-	-
36	Broiler turkeys	2	2	2	2	-	-	-	-	-
37	Broiler turkeys	3	3	3	3	-	3	-	-	-
38	Laying hens	3	3	3	3	-	3	3	-	-
39	Broiler chickens	2	2	2	2	-	-	-	-	-
40	Broiler turkeys	-	2	2	-	-	-	-	-	-
41	Broiler turkeys	3	3	3	-	-	-	-	-	-
42	Broiler chickens	3	3	3	3	-	-	-	-	-
43	Broiler turkeys	-	2	2	-	-	2	-	-	-
44	Broiler turkeys	-	2	2	-	-	-	-	-	-
45	Broiler chickens	1	1	1	1	1	-	-	-	-
46	Broiler chickens	2	2	-	-	-	-	-	-	-
47	Broiler chickens	3	3	3	3	-	-	-	-	-
48	Broiler breeding hens	3	3	3	3	-	3	3	-	-
49	Broiler chickens	2	2	2	-	-	-	-	-	-
50	Broiler chickens	-	-	-	1	-	1	-	-	-
51	Broiler breeding hens	3	3	3	3	-	3	3	-	-

52	Laying hens	2	2	2	-	-	-	-	-	-
53	Broiler breeding hens	2	2	2	2	-	2	-	-	-
54	Broiler breeding hens	1	1	1	-	-	-	-	-	-
55	Broiler breeding hens	1	1	1	1	-	1	-	-	-
56	Broiler breeding hens	3	3	3	3	-	3	3	-	-
57	Broiler chickens	-	1	1	1	-	-	-	-	-
58	Broiler chickens	3	3	3	-	-	-	-	-	-
59	Broiler turkeys	-	-	-	3	-	3	-	-	-
60	Broiler chickens	-	-	-	-	-	-	-	2	-
61	Laying hens	3	3	3	-	-	-	-	-	-
62	Broiler breeding hens	3	3	3	3	-	3	-	-	-
63	Broiler breeding hens	3	3	3	3	-	3	-	-	-
64	Broiler chickens	1	1	1	-	-	-	-	-	-
65	Broiler turkeys	-	1	1	1	-	-	-	-	-
66	Broiler chickens	2	2	2	-	2	2	-	-	-
67	Broiler chickens	3	3	3	3	3	3	-	-	-

68	Laying hens	2	-	-	-	2	-	-	-	-
69	Laying hens	2	-	-	-	2	-	-	-	-
70	Laying hens	3	3	3	3	-	3	3	-	-
71	Broiler breeding hens	3	3	3	3	-	3	3	-	-
72	Broiler chickens	3	3	3	3	3	3	-	-	-
73	Laying hens	-	3	3	-	-	-	-	-	-
74	Broiler chickens	1	1	1	-	1	-	-	-	-
75	Broiler breeding hens	3	3	3	3	-	3	3	-	-
76	Broiler breeding hens	3	3	3	3	-	3	3	-	-
77	Broiler breeding hens	3	3	3	3	-	3	3	-	-
78	Broiler breeding hens	3	3	3	3	-	3	3	-	-
79	Broiler turkeys	3	3	3	3	-	3	-	-	-
80	Broiler chickens	3	3	3	3	3	3	-	-	-
81	Broiler chickens	2	2	2	-	-	2	-	-	-
82	Broiler chickens	3	3	3	3	-	3	-	-	-
83	Broiler chickens	3	3	3	3	-	3	-	-	-

84	Broiler chickens	1	1	1	-	-	-	-	-	-
85	Broiler breeding hens	1	1	1	-	-	-	-	-	-
86	Broiler breeding hens	3	3	3	3	-	3	3	-	-
87	Broiler chickens	-	-	-	-	-	-	-	2	-
88	Broiler chickens	-	-	-	-	-	-	-	2	-
89	Broiler chickens	-	-	-	-	-	-	-	2	-
90	Broiler chickens	-	-	-	-	-	-	-	2	-
91	Broiler chickens	-	-	-	-	-	-	-	3	-
92	Laying hens	-	2	2	-	-	-	-	-	-
93	Laying hens	-	3	3	3	-	3	3	-	-
94	Broiler chickens	-	-	-	-	-	-	-	3	-
95	Broiler breeding hens	-	-	-	-	-	-	-	2	-
96	Laying hens	3	3	3	3	-	3	3	-	-
97	Broiler chickens	-	-	-	-	-	-	-	2	-
98	Broiler chickens	-	-	-	3	-	3	-	-	-
99	Broiler breeding hens	-	-	-	-	-	-	-	2	-
100	Broiler chickens	3	3	3	3	3	3	-	-	-

101	Broiler chickens	-	-	-	2	-	2	-	-	-
102	Broiler chickens	3	3	3	3	3	3	-	-	-
103	Broiler chickens	2	2	2	-	-	-	-	-	-
104	Laying hens	2	2	2	2	-	2	2	-	-
105	Broiler chickens	2	2	2	-	-	-	-	-	-
106	Broiler chickens	2	2	2	-	-	-	-	-	-
107	Laying hens	3	3	3	3	-	3	3	-	-
108	Broiler chickens	3	3	3	-	-	3	-	-	-
109	Broiler chickens	3	3	3	3	-	3	-	-	-
110	Broiler turkeys	3	3	3	-	-	-	-	-	-
111	Broiler breeding hens	-	-	-	2	-	2	-	-	-
112	Broiler chickens	3	3	3	-	-	-	-	-	-
113	Broiler chickens	1	1	1	1	-	1	-	-	-
114	Broiler breeding hens	-	-	-	-	-	-	-	3	-
115	Laying hens	1	1	-	-	-	1	-	-	-
116	Laying hens	1	1	-	-	-	-	-	-	-
117	Broiler turkeys	3	3	3	3	-	3	-	-	-
118	Broiler chickens	3	3	3	-	-	3	-	-	-

119	Broiler turkeys	3	3	3	3	-	3	-	-	-
120	Laying hens	3	3	3	3	-	3	3	-	-
121	Laying hens	-	-	-	-	-	-	-	3	-
122	Broiler chickens	2	2	2	2	2	-	-	-	-
123	Broiler chickens	2	2	2	-	2	-	-	-	-
124	Laying hens	3	3	3	3	-	3	3	-	-
125	Broiler turkeys	-	1	1	1	-	-	-	-	-
126	Broiler chickens	3	3	3	3	-	3	-	-	-
127	Broiler chickens	3	3	3	3	3	3	-	-	-
128	Broiler turkeys	3	3	3	3	-	3	-	-	-
129	Broiler turkeys	3	3	3	3	-	3	-	-	-
130	Broiler turkeys	-	1	1	-	-	-	-	-	-
131	Broiler turkeys	-	1	1	-	-	-	-	-	-
132	Laying hens	3	3	3	3	-	3	3	-	-
133	Broiler chickens	-	-	-	-	-	-	-	2	-
134	Broiler breeding hens	3	3	3	3	-	3	3	-	-
135	Broiler breeding hens	1	1	1	-	-	-	-	-	-
136	Broiler chickens	-	3	-	-	-	-	-	-	-

137	Broiler breeding hens	3	3	3	3	-	3	3	-	-
138	Broiler chickens	-	2	-	-	-	-	-	-	-
139	Broiler chickens	-	-	-	-	-	3	-	-	-
140	Broiler chickens	-	2	-	-	-	2	-	-	-
141	Broiler breeding hens	2	2	2	-	-	-	-	-	-
142	Broiler chickens	2	2	2	2	-	2	-	-	-
143	Broiler chickens	3	3	3	3	-	3	-	-	-
144	Broiler chickens	-	3	-	-	-	3	-	-	-
145	Broiler chickens	3	3	3	3	-	3	-	-	-
146	Broiler chickens	2	2	2	2	-	2	-	-	-
147	Broiler turkeys	-	2	2	-	-	-	-	-	-
148	Broiler chickens	3	3	3	-	-	-	-	-	-
149	Broiler breeding hens	2	2	2	2	-	2	2	-	-
150	Broiler turkeys	-	2	2	-	-	2	-	-	-
151	Laying hens	3	3	3	-	-	-	-	-	-
152	Broiler breeding hens	1	1	1	-	-	-	-	-	-
153	Broiler breeding	3	3	3	3	-	3	3	-	-

	hens									
154	Broiler chickens	-	-	-	-	-	-	-	3	-
155	Broiler chickens	2	2	2	-	-	-	-	-	-
156	Laying hens	-	2	2	2	-	2	2	-	-
157	Laying hens	3	3	3	3	-	3	-	-	-
158	Broiler turkeys	-	3	3	3	-	3	-	-	-
159	Laying hens	3	3	3	3	-	3	-	-	-
160	Broiler breeding hens	2	2	2	-	-	-	-	-	-
161	Broiler breeding hens	3	3	3	3	-	3	3	-	-
162	Broiler chickens	-	3	-	-	-	3	-	-	-
163	Broiler chickens	2	2	-	-	-	-	-	-	-
164	Laying hens	1	1	1	-	-	-	-	-	-
165	Broiler chickens	-	-	-	-	-	-	-	1	-
166	Broiler chickens	3	3	-	-	-	-	-	-	-
167	Broiler chickens	-	-	-	-	-	-	-	1	-
168	Broiler turkeys	2	2	2	-	-	-	-	-	-
169	Broiler chickens	-	-	-	-	-	-	-	2	-
170	Broiler chickens	-	-	-	-	-	-	-	3	-

171	Laying hens	2	2	2	2	-	2	2	-	-
172	Broiler turkeys	3	3	3	3	-	3	-	-	-
173	Broiler chickens	-	-	-	-	-	-	-	3	-
174	Broiler chickens	-	-	-	-	-	-	-	2	-
175	Laying hens	3	3	3	3	-	3	3	-	-
176	Broiler turkeys	-	3	3	-	-	-	-	-	-
177	Broiler turkeys	-	-	-	3	-	3	-	-	-
178	Broiler turkeys	2	2	2	2	-	2	-	-	-
179	Broiler turkeys	2	2	2	2	-	2	-	-	-
180	Broiler turkeys	3	3	3	-	-	-	-	-	-
181	Broiler turkeys	3	3	3	-	-	-	-	-	-
182	Broiler turkeys	3	3	3	-	-	-	-	-	-
183	Broiler turkeys	1	1	-	-	-	-	-	-	-
184	Broiler chickens	2	2	2	-	-	-	-	-	-
185	Broiler chickens	3	3	3	3	-	3	-	-	-
186	Broiler turkeys	1	1	1	-	-	-	-	-	-
187	Broiler turkeys	3	3	3	3	-	3	-	-	-
188	Broiler turkeys	3	3	-	-	-	-	-	-	-
189	Broiler turkeys	3	3	3	3	-	3	-	-	-

190	Broiler turkeys	2	2	-	-	-	-	-	-	-		
191	Broiler turkeys	1	1	-	-	-	-	-	-	-		
192	Laying hens	3	3	3	3	-	3	3	-	-		
193	Broiler turkeys	3	3	3	3	-	-	-	-	-		
194	Laying hens	1	1	1	-	-	-	-	-	-		
195	Broiler breeding hens	1	1	1	-	-	-	-	-	-		
196	Broiler breeding hens	3	3	3	3	-	3	3	-	-		
197	Broiler chickens	3	3	-	-	-	-	-	-	-		
198	Broiler chickens	-	1	-	-	-	-	-	-	-		
199	Broiler turkeys	3	3	3	3	-	3	-	-	-		
200	Broiler chickens	2	2	2	2	-	2	-	-	-		
201	Laying hens	1	1	1	-	-	-	-	-	-		
202	Broiler turkeys	2	2	-	2	-	2	-	-	-		
203	Broiler chickens	2	2	2	-	-	-	-	-	-		
204	Broiler chickens	2	2	2	-	-	-	-	-	-		
205	Broiler turkeys	1	1	1	1	-	1	-	-	-		
206	Broiler breeding hens	3	3	3	3	-	3	3	-	-		

207	Broiler chickens	3	3	3	-	-	-	-	-	-
208	Laying hens	1	1	1	-	-	-	-	-	-
209	Broiler breeding hens	3	3	3	3	-	3	3	-	-
210	Broiler breeding hens	3	3	3	3	-	3	3	-	-
211	Broiler breeding hens	3	3	3	3	-	3	3	-	-
212	Broiler breeding hens	3	3	3	3	-	3	3	-	-
213	Broiler chickens	3	3	3	3	3	3	-	-	-
214	Broiler chickens	3	3	3	3	3	3	-	-	-
215	Broiler turkeys	-	-	-	3	-	3	-	-	-
216	Broiler chickens	2	2	2	2	2	2	-	-	-
217	Broiler turkeys	3	3	3	3	-	3	-	-	-
218	Broiler turkeys	1	1	-	-	-	-	-	-	-
219	Broiler chickens	3	3	3	3	-	3	-	-	-
220	Broiler turkeys	3	3	3	3	-	3	-	-	-
221	Broiler turkeys	3	3	3	3	-	3	-	-	-
222	Broiler turkeys	3	3	3	3	3	3	-	-	-
223	Broiler breeding	3	3	3	3	-	3	-	-	-

	hens									
224	Broiler turkeys	3	3	3	3	3	3	-	-	-
225	Broiler breeding hens	3	3	3	3	-	3	-	-	-
226	Broiler breeding hens	3	3	3	3	-	3	-	-	-
227	Broiler breeding hens	3	3	3	3	-	3	-	-	-
228	Broiler chickens	-	-	3	3	3	3	-	-	-
229	Broiler turkeys	3	3	3	3	3	3	-	-	-
230	Broiler turkeys	3	3	3	3	3	3	-	-	-
231	Broiler turkeys	3	3	3	3	3	3	-	-	-
232	Broiler turkeys	3	3	3	3	3	3	-	-	-
233	Broiler turkeys	3	3	3	3	3	3	-	-	-
234	Broiler turkeys	3	3	3	3	3	3	-	-	-
235	Broiler turkeys	3	3	3	3	3	3	-	-	-
236	Broiler turkeys	3	3	3	3	3	3	-	-	-
237	Broiler turkeys	3	3	3	3	-	3	-	-	-
238	Broiler turkeys	1	1	-	-	-	-	-	-	-
239	Broiler breeding hens	3	3	3	3	-	3	3	-	-

240	Broiler breeding hens	3	3	3	3	-	3	3	-	-
241	Broiler breeding hens	3	3	3	3	-	3	3	-	-
242	Broiler turkeys	-	-	-	3	-	3	-	-	-
243	Broiler chickens	2	2	2	-	-	-	-	-	-
244	Broiler chickens	3	3	3	-	-	-	-	-	-
245	Broiler chickens	3	3	3	3	-	3	-	-	-
246	Laying hens	3	3	3	3	-	3	3	-	-
247	Laying hens	-	3	3	3	-	3	-	-	-
248	Broiler breeding hens	2	2	2	-	-	-	-	2	-
249	Broiler chickens	2	2	2	-	-	-	-	-	-
250	Broiler chickens	-	-	-	-	-	-	-	3	-
251	Broiler chickens	-	-	-	-	-	-	-	3	-
252	Broiler chickens	2	2	2	2	2	2	-	-	-
253	Broiler chickens	-	-	-	-	-	-	-	1	-
254	Broiler chickens	-	-	-	-	-	-	-	2	-
255	Broiler chickens	-	-	-	-	-	-	-	1	-
256	Broiler chickens	3	3	3	3	-	-	-	-	-

257	Broiler breeding hens	-	-	-	-	-	-	-	2	-
258	Broiler chickens	-	-	-	-	-	-	-	2	-
259	Broiler chickens	-	-	-	-	-	-	-	3	-
260	Broiler chickens	-	-	-	-	-	-	-	2	-
261	Broiler chickens	-	-	-	-	-	-	-	2	-
262	Broiler chickens	-	-	-	-	-	-	-	1	-
263	Broiler chickens	-	-	-	-	-	-	-	2	-
264	Broiler turkeys	2	2	2	-	-	-	-	-	-
265	Broiler chickens	1	1	1	1	1	-	-	-	-
266	Broiler turkeys	-	3	3	-	-	-	-	-	-
267	Broiler turkeys	3	3	3	3	-	3	-	-	-
268	Broiler chickens	3	3	3	3	3	3	3	-	-
269	Broiler chickens	3	3	3	3	3	3	3	-	-
270	Broiler chickens	3	3	3	3	3	3	3	-	-
271	Broiler breeding hens	3	3	3	3	-	3	3	-	-
272	Broiler turkeys	3	3	3	3	-	3	-	-	-
273	Broiler turkeys	3	3	3	3	-	3	-	-	-
274	Broiler turkeys	3	3	3	3	-	3	-	-	-

275	Broiler turkeys	-	-	-	3	-	3	-	-	-
276	Broiler turkeys	3	3	3	3	-	3	-	-	-
277	Broiler turkeys	3	3	3	-	-	-	-	-	-
278	Broiler breeding hens	3	3	3	3	-	3	3	-	-
279	Broiler breeding hens	3	3	3	3	-	3	3	-	-
280	Broiler breeding hens	3	3	3	3	-	3	3	-	-
281	Broiler breeding hens	3	3	3	3	-	3	3	-	-
282	Broiler breeding hens	3	3	3	3	-	3	3	-	-
283	Broiler breeding hens	3	3	3	3	-	3	3	-	-
284	Broiler breeding hens	3	3	3	3	-	3	3	-	-
285	Broiler breeding hens	3	3	3	3	-	-	-	-	-
286	Broiler turkeys	-	3	3	3	3	3	-	-	-
287	Laying hens	2	2	2	-	-	-	-	-	-
288	Broiler breeding hens	3	3	3	3	-	3	3	-	-

289	Broiler breeding hens	3	3	3	3	-	3	3	-	-
290	Broiler chickens	3	3	3	3	3	3	3	-	-

Legend:

1 – Group 1 of pathological lesions; two or more necrotic foci located in the internal organs (liver, spleen, kidney, pericardial sac), cloudiness of the air sacs, hyperaemia of the yolks (in laying hens), hyperaemia in the navel area and yolk sac (in the case of chicks).

2 - Group 2 of pathological lesions; a thin layer of fibrous exudate located on the serosa and internal organs (liver, spleen, kidney, pericardial sac, air sacs), a cheese-like coating on the yolks, cloudy fluid in the oviduct (in layers), altered hyperaemia of the yolk sac (in chicks).

3 - Group 3 of pathological lesions; a thick layer of fibrous exudate on the surface of internal organs (liver, spleen, kidney, pericardial sac, air sacs) and serous membranes, coagulated eggs in the body cavity (in layers), inflammatory lesions in the umbilical region and clotted masses in the yolk sac (in chicks).

„-„ no pathological lesions
