# Supplementary figures and images for: Molecular and Serological Characteristics of Avian Pathogenic Escherichia coli Isolated from Various Clinical Cases of Poultry Colibacillosis in Poland
Source: Animals (Basel). 2022 Apr 22;12(9):1090. doi: 10.3390/ani12091090 (PMC9106014; doi:10.3390/ani12091090)

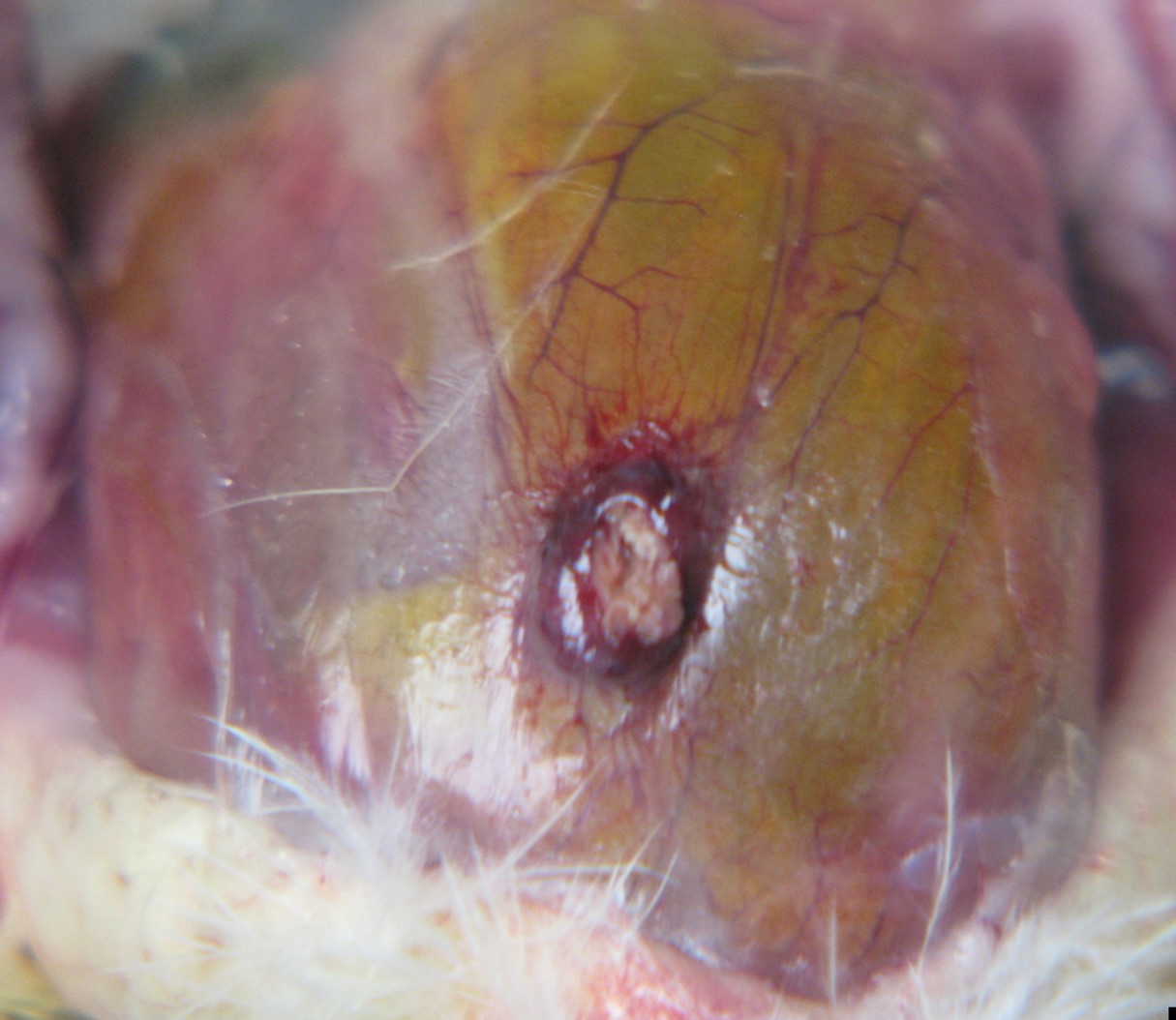

Supplement: Supplementary file 1 [file animals-12-01090-s001.zip › Figure S1.jpg]

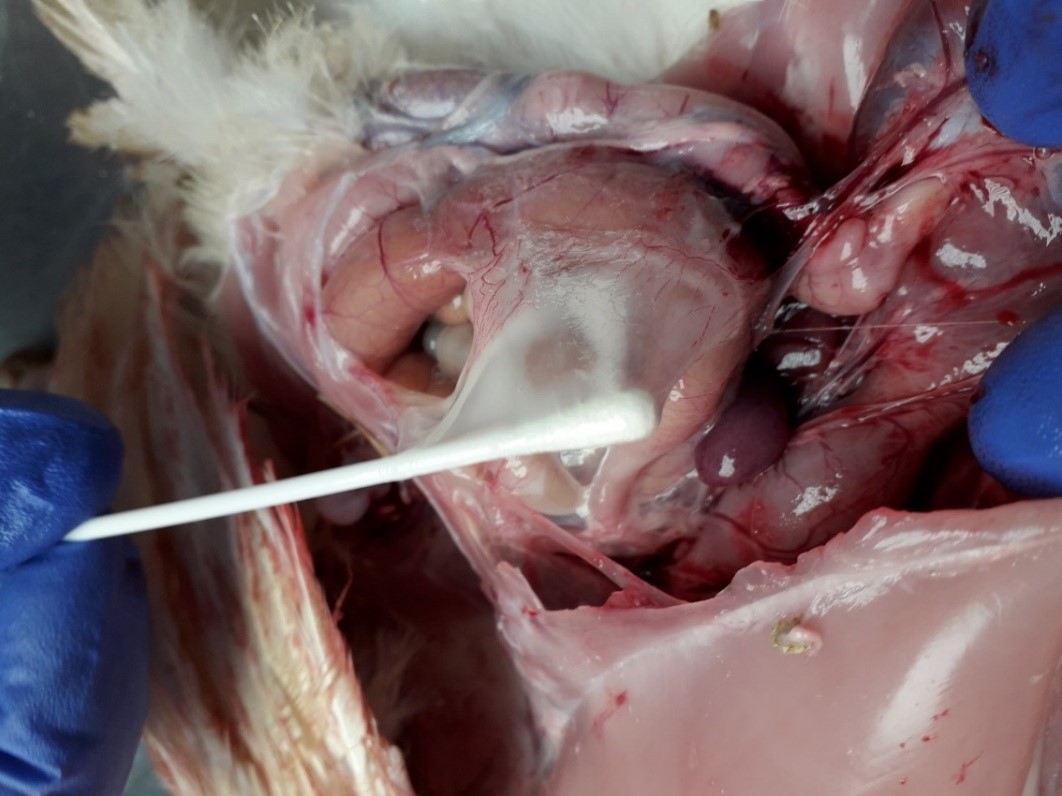

Supplement: Supplementary file 1 [file animals-12-01090-s001.zip › Figure S2.jpg]

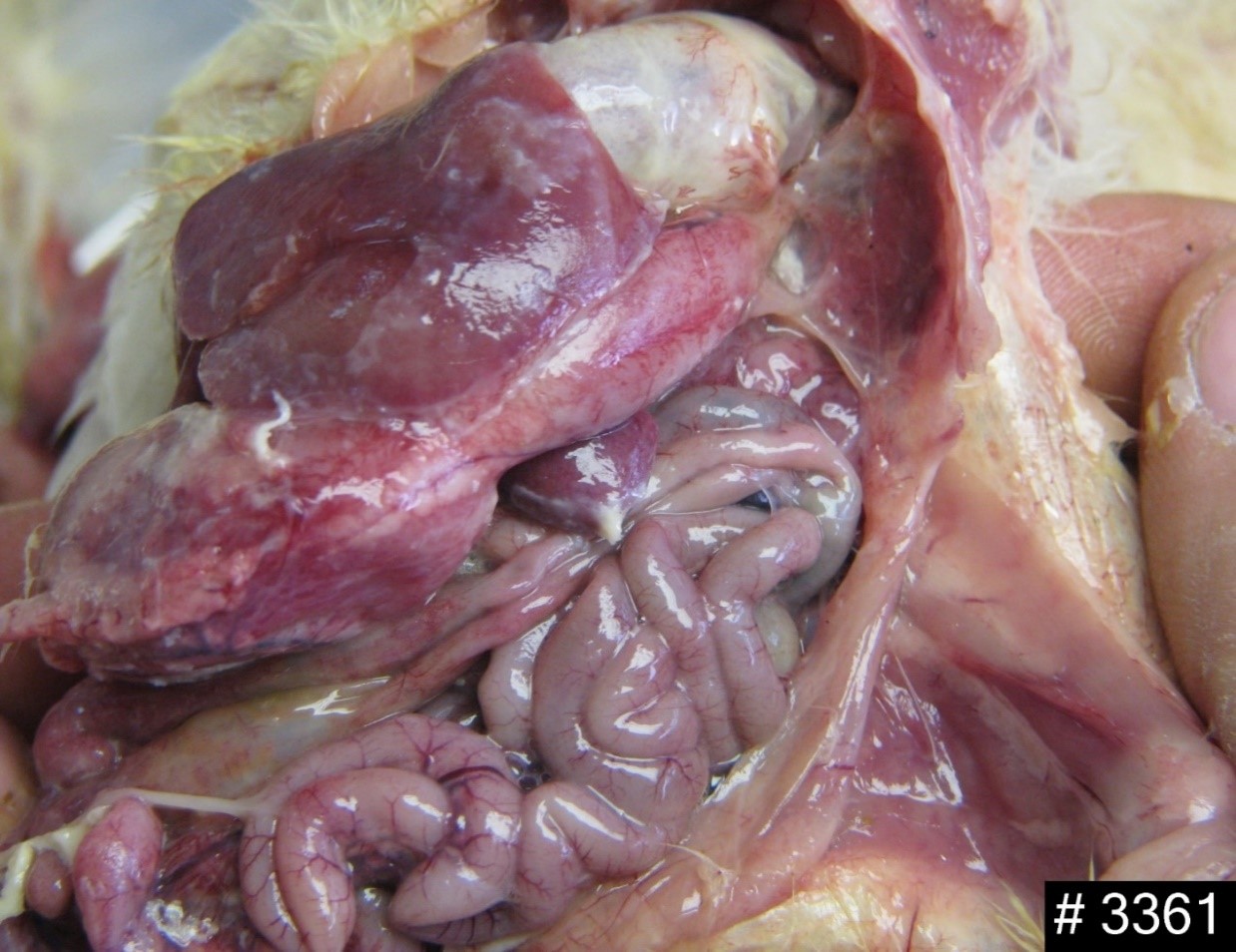

Supplement: Supplementary file 1 [file animals-12-01090-s001.zip › Figure S3.jpg]

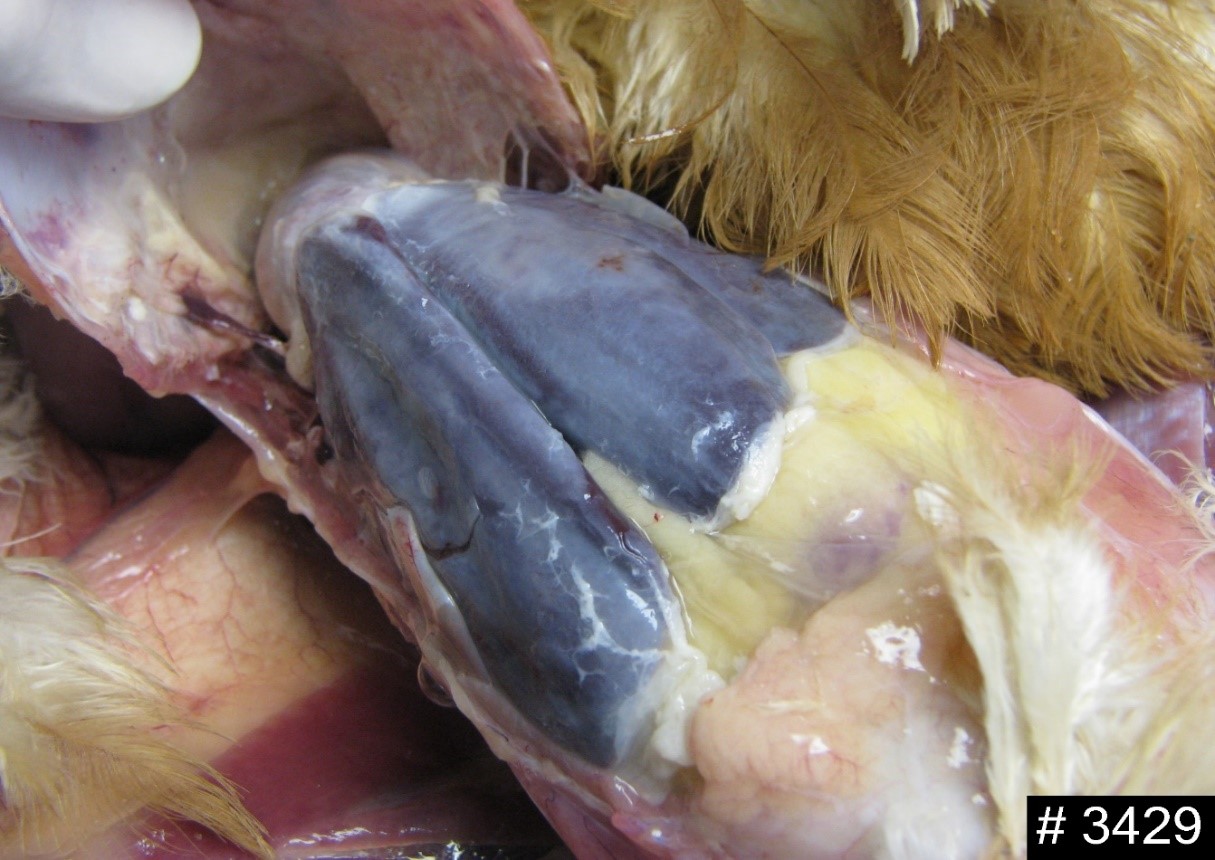

Supplement: Supplementary file 1 [file animals-12-01090-s001.zip › Figure S4.jpg]

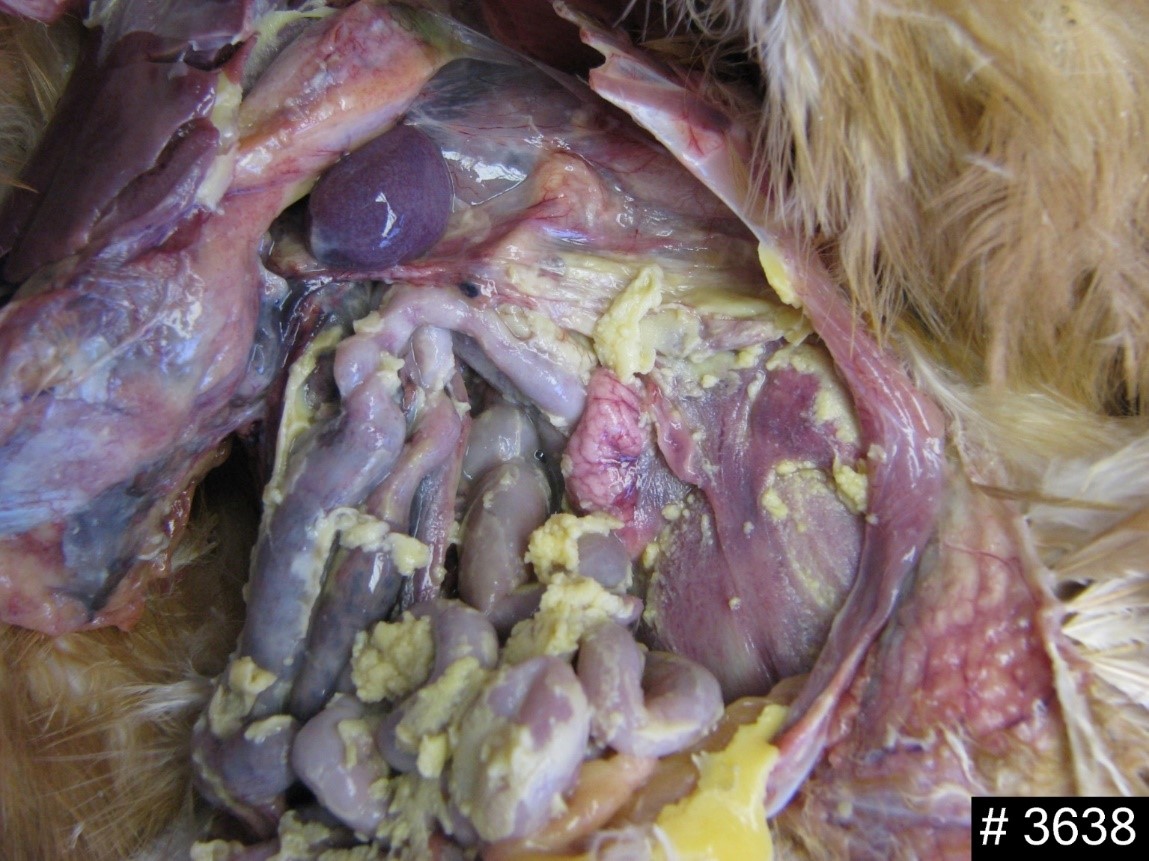

Supplement: Supplementary file 1 [file animals-12-01090-s001.zip › Figure S5.jpg]

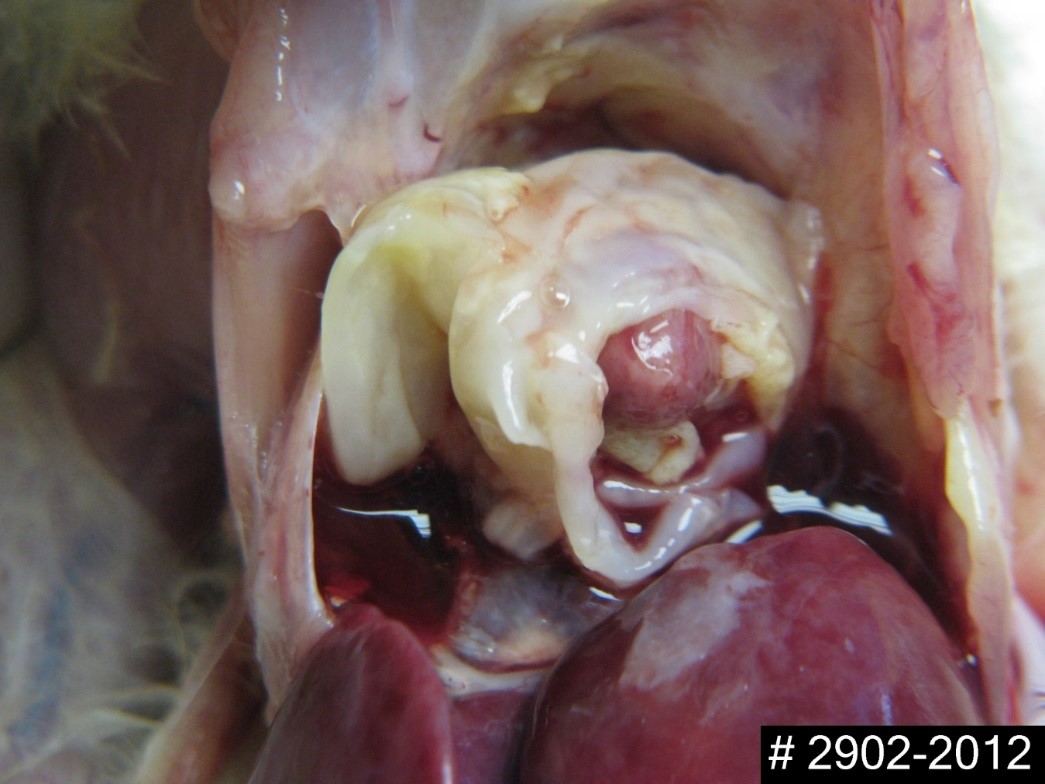

Supplement: Supplementary file 1 [file animals-12-01090-s001.zip › Figure S6.jpg]
